# IL-10 Gene-Modified Human Amniotic Mesenchymal Stem Cells Augment Regenerative Wound Healing by Multiple Synergistic Effects

**DOI:** 10.1155/2019/9158016

**Published:** 2019-06-11

**Authors:** Shune Xiao, Guangtao Huang, Zairong Wei, Kaiyu Nie, Zhiyuan Liu, Chengliang Deng, Dali Wang

**Affiliations:** Department of Plastic Surgery, Affiliated Hospital of Zunyi Medical University, Zunyi, Guizhou, China

## Abstract

Mesenchymal stem cells (MSCs) possess a capacity to enhance cutaneous wound healing that is well characterized. However, the therapeutic effect of MSCs appears to be limited. Modifying MSC target genes to increase necessary biological effects is a promising strategy for wound therapy. Interleukin-10 (IL-10) is an anti-inflammatory cytokine that has a therapeutic effect on wound healing. In this study, we modified human amniotic mesenchymal stem cells (hAMSCs) using recombinant lentiviral vectors for expressing IL-10 and evaluated the therapeutic effects of hAMSCs-IL-10 in wound healing. We elucidated the mechanisms underlying the effects. We found that promoting wound healing was maintained by synergistic effects of hAMSCs and IL-10. hAMSCs-IL-10 showed stronger biological effects in accelerating wound closure, enhancing angiogenesis, modulating inflammation, and regulating extracellular matrix remodeling than hAMSCs. hAMSCs-IL-10 would be better at promoting wound healing and improving healing quality. These data may provide a theoretical foundation for clinical administration of hAMSCs-IL-10 in cutaneous wound healing and skin regeneration.

## 1. Introduction

Wound healing is a complex process that includes inflammation, cell proliferation, angiogenesis, and extracellular matrix (ECM) remodeling [1]. Since scar-free regenerative wound healing of human fetuses was reported, efforts have been directed at investigating the underlying mechanisms by comparing the wound-healing processes of scarless and scarring wounds in multiple animal models. A key difference identified in fetal wound healing is a low inflammatory reaction compared to postnatal wounds. Interleukin- (IL-) 10 is essential for the ability of fetal wounds to have low inflammatory reactions for scarless regenerative wound healing [1, 2]. Misalignment of biodynamic processes can lead to delayed healing and excessive scarring, which present large challenges to healthcare systems globally.

Mesenchymal stem cells (MSCs) are widely reported to have an active function in the process of wound healing [3]. MSC-based skin engineering combined with genetic recombination in which MSCs are the seed cells and the vehicle for gene delivery to the wound site represents the most promising option for a strategy for wound therapy [4]. Alapure et al. found that bone marrow MSCs with incorporated biomaterial covering burn wounds promote closure, reepithelialization, granulation tissue formation, and vascularization of burn wounds [5]. Modification of MSCs by hepatocyte growth factor and vascular endothelial growth factor (VEGF) genes to increase necessary biological effects and augment wound healing has been confirmed [6, 7].

IL-10 is an anti-inflammatory and antifibrotic cytokine. It is essential for the ability of a fetus to heal regeneratively [1, 2]. IL-10 has been shown to recapitulate scarless regenerative healing in postnatal tissue through pleiotropic effects. Besides regulating the inflammatory response, IL-10 has novel functions as a regulator of the extracellular matrix, fibroblast cellular function, and endothelial progenitor cells [8–10]. Given this information, we hypothesized that overexpression of IL-10 in MSCs may have beneficial effects on MSCs facilitating regenerative wound healing and preventing scar formation. In this study, we evaluated the therapeutic effects of IL-10 gene-modified hAMSCs (hAMSCs-IL-10) on anti-inflammation and antifibrosis effects and promotion of wound healing.

## 2. Materials and Methods

### 2.1. Animals and Ethics Approval

Wild-type, 7- to 8-week-old C57BL/6 mice were provided by the Animal Experimental Center of the Army Military Medical University (Chongqing, China). Human placentas were obtained from donors following normal or cesarean deliveries after obtaining informed consent and approval from the Affiliated Hospital of Zunyi Medical University Institutional Review Board. All experimental procedures were performed in accordance with the guidelines and regulations established by the Medical Ethics Committee of Zunyi Medical University (Zunyi, China).

### 2.2. Isolation, Culture, and Flow Cytometry Identification of hAMSCs

hAMSCs were isolated and cultured as previously described, with slight modifications [11]. The amnion was separated from the chorion mechanically and rinsed three times in phosphate-buffered saline (PBS) with 1% penicillin-streptomycin (Gibco, Carlsbad, CA, USA). The amnion was cut into small pieces and incubated with 0.25% trypsin/EDTA (0.05%, Gibco) at 37°C for 40 min to remove amniotic epithelial cells. After rinsing with PBS, amnion fragments were minced and digested with 0.75 mg/mL collagenase II (Sigma-Aldrich, St. Louis, MO, USA) at 37°C for 90 min with gentle shaking. An equal volume of Dulbecco's modified Eagle's medium (DMEM, Gibco) supplemented with 10% fetal bovine serum (FBS, Gibco) was added to stop the enzymatic reaction, and cell suspensions were filtered with 100 *μ*m mesh cell strainers. Cell suspensions were centrifuged at 500 ×*g* for 5 min, and cell pellets were resuspended and cultured in DMEM/F12 medium (Gibco) supplemented with 10% FBS and 1% penicillin-streptomycin. At 80% confluence, hAMSCs were subcultured, and cells at passage 3 were used in following experiments.

Flow cytometry was used to identify characteristics of hAMSCs and detect stem cell-related cell surface markers. For flow cytometry, 1 × 10^6^ cells/100 *μ*L were collected and stained with antibodies against CD90 (FITC, clone: 5E10), CD105 (PerCP-Cy5.5, clone: 266), CD73 (APC, clone: AD2), CD44 (PE, clone: G44-26), CD34 (PE, clone: 581), CD11b (PE, clone: ICRF44), CD19 (PE, clone: HIB19), CD45 (PE, clone: HI30), HLA-DR (PE, clone: G46-6), mIgG1 (*κ* FITC, clone: X40), mIgG1 (*κ* PerCP-Cy5.5, clone: X40), mIgG1 (*κ* APC, clone: X40), mIgG1 (*κ* PE, clone: X40), mIgG2a (*κ* PE, clone: G155-178), and mIgG2b (*κ* clone: 27-35). All antibodies were used at 1 : 1000 (BD, Pharmingen, USA). Samples were incubated at room temperature for 30 minutes, washed with PBS, and analyzed with a MoFlo XDP flow cytometer (Beckman Coulter, Brea, CA) with Kaluza software (Beckman Coulter).

### 2.3. Osteogenesis and Adipogenesis of hAMSCs

Multilineage differentiation of hAMSCs was tested. To induce differentiation into osteocytes and adipocytes, cells were cultured in osteocyte differentiation medium or adipocyte differentiation medium. After 14 days of differentiation, cells were stained with Alizarin Red S (Cyagen, Guangzhou, China) or Oil Red O (Cyagen).

### 2.4. Construction and Characterization of IL-10-Modified hAMSCs

An IL-10-overexpressing vector (carrying green fluorescent protein) was from Shanghai Innovation Biotechnology Co. Ltd. The multiplicity of infection (MOI) was 30. Lentiviruses used in this study were LV-IL-10, a replication-defective lentivirus expressing IL-10, and LV-Null, a replication-defective lentivirus not carrying any exogenous genes. The hAMSCs were transfected with 30 MOI of LV-IL-10 (hAMSCs-IL-10) or LV-Null (hAMSCs-Null) and observed under a fluorescence microscope at 48 h postinfection. Expression of IL-10 in supernatants of hAMSCs-IL-10, hAMSCs-Null, and hAMSCs was detected by the enzyme-linked immunosorbent assay (ELISA).

### 2.5. *In Vivo* Animal Studies

A full-thickness skin defect model was generated. C57BL/6 mice were anaesthetized by intraperitoneal injection of 10 g/L pentobarbital sodium (0.4 mL/100 g), and the dorsa were shaved, depilated, and cleaned with betadine. Two full-thickness excisional wounds (1 cm^2^) were made symmetrically on both sides of the midline of the back. Mice were randomized into four groups and injected subcutaneously around the wound area with saline as a control (100 *μ*L, *n* = 10), IL-10-hAMSCs (1 × 10^6^ cells/100 *μ*L saline, *n* = 10), hAMSCs-Null (1 × 10^6^ cells/100 *μ*L saline, *n* = 10), or hAMSCs (1 × 10^6^ cells/100 *μ*L saline, *n* = 10). Wound healing was evaluated on the basis of gross observation at days 1, 3, 7, and 14 after treatment, and the wound healing rate was calculated.

### 2.6. Inflammatory Response

To evaluate the anti-inflammatory effect of IL-10-hAMSCs, skin around wounds was collected at 3, 7, and 14 d after treatment, and inflammatory cell infiltration was observed by hematoxylin and eosin (H&E) staining. Expression levels of inflammatory factors IL-10, tumor necrosis factor-*α* (TNF-*α*), and IL-6 were quantitatively assayed with an ELISA assay kit (Sigma-Aldrich) following the manufacturer's instructions. The real-time fluorescence quantification polymerase chain reaction (qPCR) was used to detect relative mRNA levels for IL-10, TNF-*α*, and IL-6. For qPCR, total RNA was extracted from tissue around wounds using the RNAiso Plus reagent (Takara, Dalian, China); cDNA was synthesized from 2 *μ*g total RNA with SYBR PrimeScript RT-PCR Kits (Takara), and qPCR was carried out using SYBR PrimeScript RT-PCR Kits on a Stratagene MX3005P qPCR system (Agilent Technologies, Santa Clara, CA, USA). All steps were performed according to the manufacturer's protocol. Fold change of each target gene was normalized to glyceraldehyde 3-phosphate dehydrogenase (*GAPDH*) mRNA.

### 2.7. Evaluation of Vascularization

To observe angiogenesis during wound healing, skin around wounds was collected, and microvessels were assayed by tissue H&E staining. Expression of angiogenic factors, VEGF, and basic fibroblast growth factor (bFGF) was detected with ELISA. For ELISA, the concentrations of VEGF and bFGF were quantitatively measured with a Quantikine enzyme-linked immunosorbent assay kit (Sigma-Aldrich) following the manufacturer's instructions.

### 2.8. Evaluation of Extracellular Matrix Remodeling

To explore the effect of IL-10-hAMSCs on ECM production and remodeling during the wound healing process, wound skin was collected, and collagen was assayed by Masson trichrome staining according to the manufacturer's instruction. Collagen content was calculated by ImageJ software as the percentage of stained area to total area of the section. Expression of transforming growth factor-*β* (TGF-*β*), matrix metalloprotein-1 (MMP-1), and tissue inhibitor of metalloprotein-1 (TIMP-1) was measured with ELISA and immunohistochemical staining. For immunohistochemical staining, antibodies were against TGF-*β* (1 : 150, Abcam), MMP-1 (1 : 200, Abcam), and TIMP-1 (1 : 250, Abcam).

### 2.9. *In Vivo* Tracing

To follow the fate of hAMSCs *in vivo*, untransfected hAMSCs were labeled with a cell tracker CM-DiI (Invitrogen, Grand Island, NY, USA) prior to grafting, according to the manufacturer's instructions. hAMSCs-IL-10 and hAMSCs-Null expressed green fluorescence protein. On day 14, wound skin was harvested, and frozen sections were prepared. Survival of cells was observed under a fluorescent microscopic system (IX71 FL, Olympus, Japan).

### 2.10. Statistical Analysis

Data are expressed as the mean ± standard deviation (SD). One-way analysis of variance was performed for comparison between different groups using SPSS 17.0 software (IBM, Armonk, NY, USA). Differences with *P* < 0.05 were regarded as statistically significant.

## 3. Results

### 3.1. Characterization of hAMSCs

**Figure 1:**
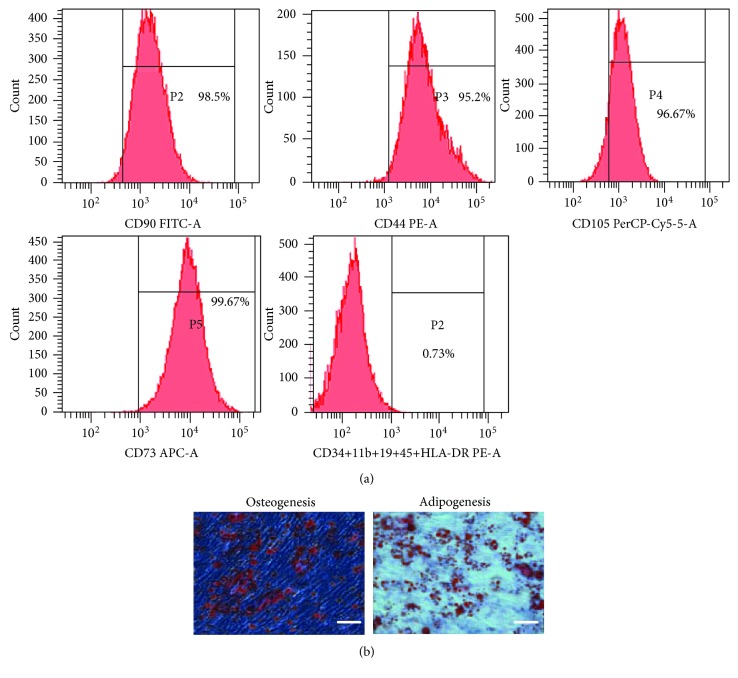
Characterization of hAMSCs. (a) FACS analysis of cell markers of hAMSCs. hAMSCs were >90% positive for CD105, CD73, CD44, and CD90 and negative for CD34, CD11b, CD19, CD45, and HLA-DR. (b) Differentiation of hAMSCs into osteocytes and adipocytes. Cells cultured under osteogenic or adipogenic culture conditions were stained for calcium deposits with Alizarin red staining or lipid droplets with Oil Red O staining. Scale bar = 50 *μ*m. hAMSCs: human amniotic mesenchymal stem cells.

Flow cytometry showed expression of surface markers of hAMSCs. hAMSCs were >90% positive for CD105, CD73, CD44, and CD90 and negative for CD34, CD11b, CD19, and CD45 (Figure 1(a)). In addition, hAMSCs were negative for HLA-DR, indicating that they possessed low immunogenicity (Figure 1(a)). hAMSCs differentiated into osteocytes as demonstrated by positive Alizarin Red staining and adipocytes as shown by Oil Red O staining (Figure 1(b)). These results indicated that cultured hAMSCs possess stem cell characteristics.

### 3.2. Transfection Efficiency and IL-10 Expression of hAMSCs

**Figure 2:**
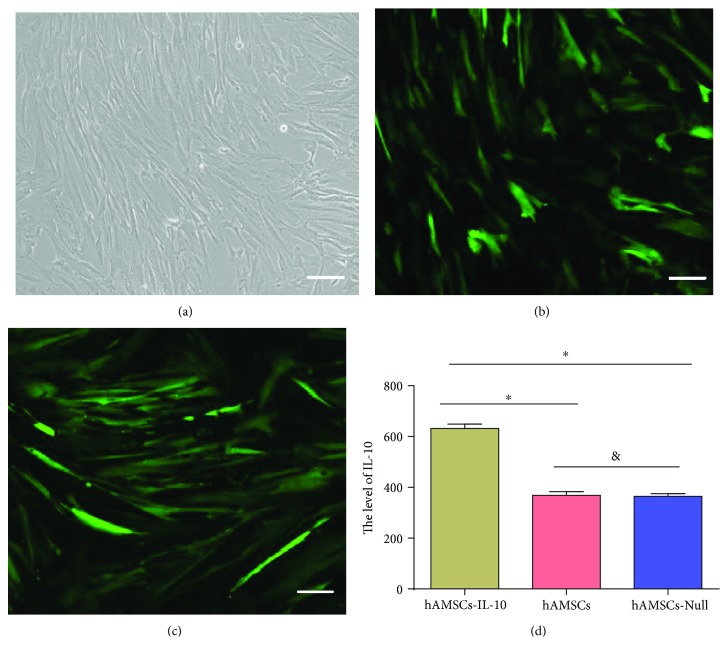
Morphology, transfection efficiency, and IL-10 expression of hAMSCs. (a) Phase-contrast micrograph of hAMSCs showing spindle-shaped morphology. (b) Fluorescence micrographs of hAMSCs after LV-IL-10 infection for 48 h. (c) Fluorescence micrographs of hAMSCs after LV-Null infection for 48 h. (d) IL-10 expression of hAMSCs; the concentration of IL-10 from hAMSCs-IL-10 increased compared to that of hAMSCs-Null and hAMSCs. ^∗^*P* < 0.05 and ^&^*P* > 0.05. LV-IL-10: replication-defective lentivirus expressing IL-10; LV-Null: replication-defective lentivirus not carrying any exogenous genes. Scale bar = 500 *μ*m.

Cultured hAMSCs were spindle-shaped with a relatively high nucleus-to-cytoplasm ratio (Figure 2(a)). After infection with lentivirus (LV-IL-10 and LV-Null), hAMSCs were observed under a fluorescence microscope at 48 h postinfection. The cells showed a uniform spindle shape and a high transfection rate of 90% (Figures 2(b) and 2(c)). We detected levels of IL-10 in the supernatant by ELISA at 48 h after infection of hAMSCs. Expression of IL-10 from hAMSCs-IL-10 increased compared to hAMSCs-Null and hAMSCs (*P* < 0.05) (Figure 2(d)).

### 3.3. IL-10 Promoted Wound Recovery

**Figure 3:**
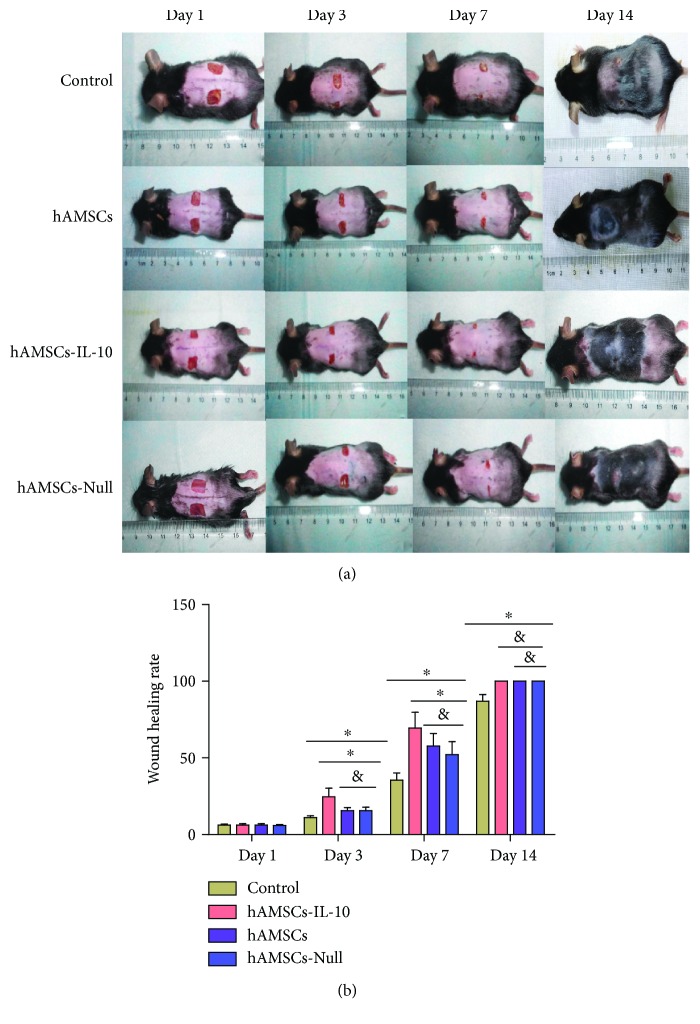
Injection of hAMSCs-IL-10 into subdermal periwound edges promoted wound healing. (a) Representative images of wound healing at indicated time points after cell transplantation. (b) Histogram of the healing rate of wounds at indicated time points with statistical analysis. Wound healing in the hAMSCs-IL-10 group was more rapid than that in the other three groups. ^∗^*P* < 0.05 and ^&^*P* > 0.05.

The complete healing of cutaneous wounds with good epithelization was evaluated by general observation. Wound healing in the hAMSCs-IL-10 group was more rapid than in the other three groups. At days 3 and 7 after cell transplantation, the hAMSCs-IL-10, hAMSCs-Null, and hAMSC groups showed significantly higher wound healing than the control. The hAMSCs-IL-10 group had significantly smaller wound sizes than the other groups between days 3 and 7. On day 14, the hAMSCs-IL-10, hAMSCs-Null, and hAMSC groups achieved complete wound healing, whereas the control group still had unhealed wounds (Figures 3(a) and 3(b)).

### 3.4. IL-10-hAMSCs Had Anti-inflammatory Effects during the Healing Process

**Figure 4:**
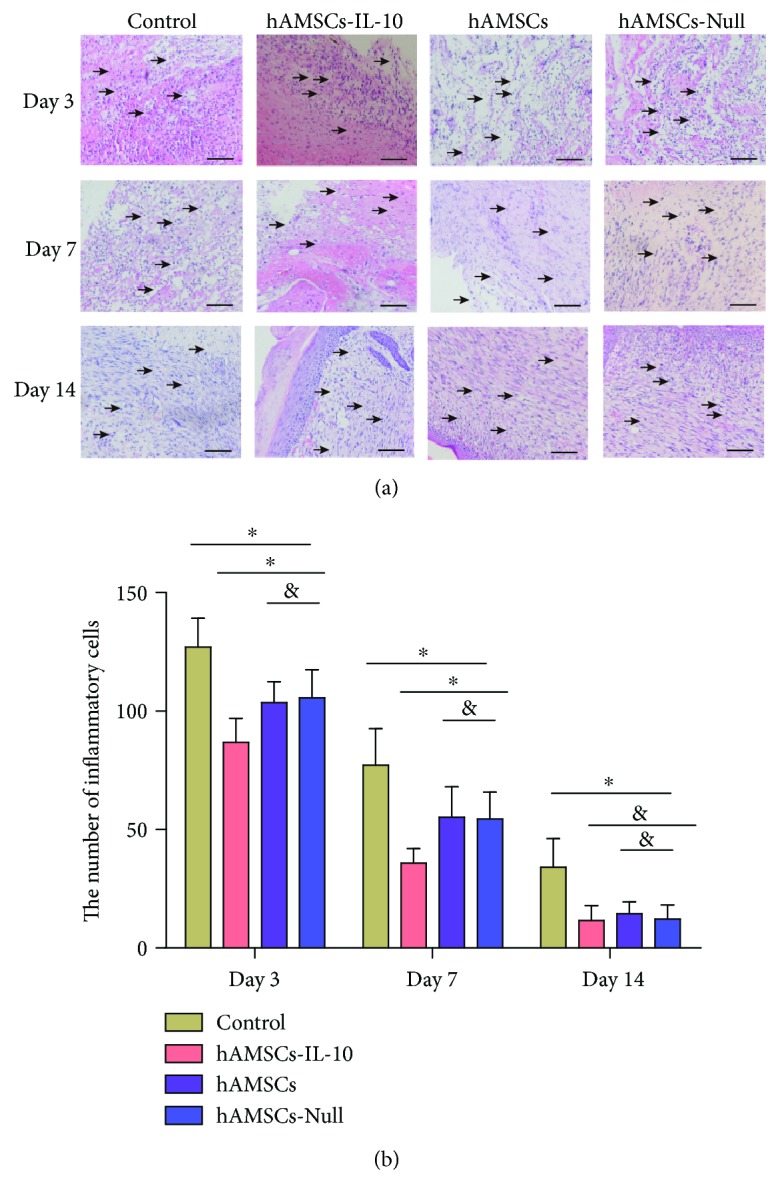
Inflammatory cell infiltration of periwounds. (a) Histology of inflammatory cell infiltration in the dermis, showing decrease in hAMSC-treated groups on days 3, 7, and 14 compared with controls. The hAMSCs-IL-10 group had the lowest infiltration of inflammatory cells. No significant difference was seen between the hAMSCs-Null and hAMSC groups. Arrows indicate inflammatory cells. (b) Quantification of inflammatory cells in the dermis by groups at indicated time points. Infiltration of inflammatory cells into the dermis decreased after treatment with hAMSCs-IL-10 compared with hAMSCs, hAMSCs-Null, or control on days 3 and 7 and decreased with hAMSCs and hAMSCs-Null compared with the control. No significant differences among the cell groups were seen in inflammatory cell infiltration on day 14. ^∗^*P* < 0.05 and ^&^*P* > 0.05. Scale bar = 500 *μ*m.

H&E staining of periwound skin sections revealed a large number of inflammatory cells infiltrating in all groups on day 3. The total number of inflammatory cells in the hAMSCs-IL-10 group was significantly lower than in the other three groups. On day 7, the number of inflammatory cells in the hAMSCs-IL-10, hAMSCs-Null, and hAMSC groups was reduced; the hAMSCs-IL-10 group showed the fewest inflammatory cells infiltrating, and many inflammatory cell infiltrations were seen in the control group. On day 14, the control group had little inflammatory cell infiltration, and almost no inflammatory cells were observed in the hAMSCs-IL-10, hAMSCs-Null, and hAMSC groups (Figures 4(a) and 4(b)).

**Figure 5:**
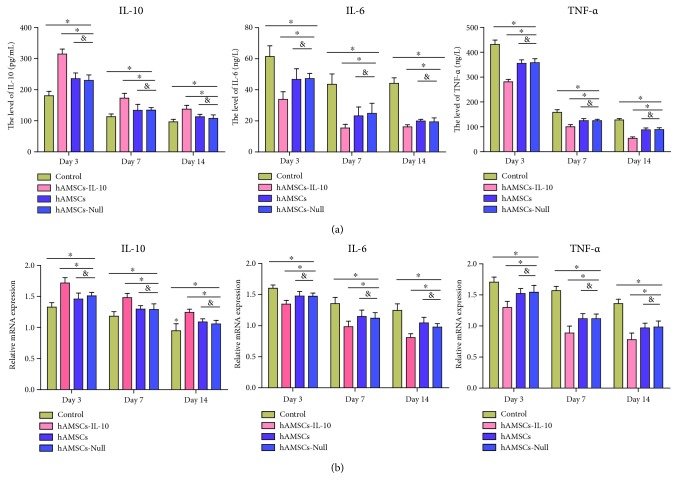
Expression of inflammatory factors. (a) ELISA of inflammatory factor expression by groups at indicated time points. The hAMSCs-IL-10 group showed the lowest expression of proinflammatory factors IL-6 and TNF-*α* and the highest expression of anti-inflammatory cytokine IL-10 on days 3, 7, and 14 compared with the hAMSCs, hAMSCs-Null, and control groups. Expression of proinflammatory factors IL-6 and TNF-*α* decreased after treatment with hAMSCs or hAMSCs-Null compared with the control on days 3, 7, and 14. Expression of anti-inflammatory cytokine IL-10 increased after treatment with hAMSCs or hAMSCs-Null compared with the control on days 3, 7, and 14. No significant differences among the hAMSCs and hAMSCs-Null groups were seen in expression of inflammatory factors (*n* = 3). (b) qPCR of inflammatory factor expression, in accord with ELISA results (*n* = 3). ^∗^*P* < 0.05 and ^&^*P* > 0.05.

We detected levels of the main anti-inflammatory cytokine IL-10 and proinflammatory factors IL-6 and TNF-*α* by ELISA and qPCR. The anti-inflammatory cytokine and proinflammatory factors peaked on day 3 and gradually decreased thereafter (Figure 5(a)). The level of IL-10 in the hAMSCs-IL-10 group was significantly higher than in the other three groups. The levels of IL-6 and TNF-*α* in the hAMSCs-IL-10 group were significantly lower than in the other three groups (Figure 5(a)). The levels of IL-6 and TNF-*α* were lower, and that of IL-10 was higher in the hAMSCs and hAMSCs-Null groups compared with controls on days 3, 7, and 14. No significant differences between the hAMSCs and hAMSCs-Null groups were seen in expression of inflammatory factors. The expression of IL-10, IL-6, and TNF-*α* was confirmed by qPCR (Figure 5(b)). The expression of the relative mRNA levels of IL-10, IL-6, and TNF-*α* was consistent with the ELISA results.

### 3.5. IL-10-hAMSCs Upregulated Expression of Angiogenic Factors and Promoted Angiogenesis

**Figure 6:**
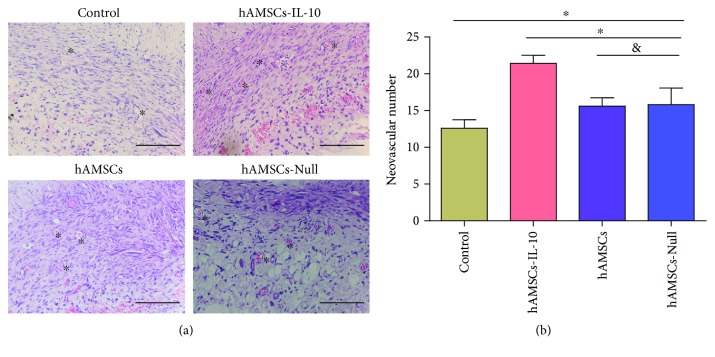
Effects of hAMSCs on wound neovascularization on day 7 after cell transplantation. (a) H&E staining showed more new blood vessels in the hAMSCs-IL-10, hAMSCs, and hAMSCs-Null groups than in the control group. The hAMSCs-IL-10 group had the most new blood vessels, and the hAMSCs-Null and hAMSC groups were not significantly different. ^∗^New blood vessel. Scale bar = 500 *μ*m. (b) Quantification of new blood vessels in the dermis by groups on day 7. The number of new blood vessels in the dermis increased after treatment with hAMSCs-IL-10 compared with hAMSCs, hAMSCs-Null, or control on day 7 and increased with hAMSCs and hAMSCs-Null compared with the control. No significant differences between the hAMSCs and hAMSCs-Null groups were seen in vascularization on day 7. ^∗^*P* < 0.05 and ^&^*P* > 0.05.

At day 7 after cell transplantation, wound neovascularization occurred in all four groups. Neovascularization was quantified by counting microvessels. Compared to the control group, the hAMSCs-IL-10, hAMSCs-Null, and hAMSC groups had more microvessels. Also, the number of microvessels in the hAMSCs-IL-10 group was significantly higher than that in the hAMSCs-Null and hAMSC groups (Figures 6(a) and 6(b)).

**Figure 7:**
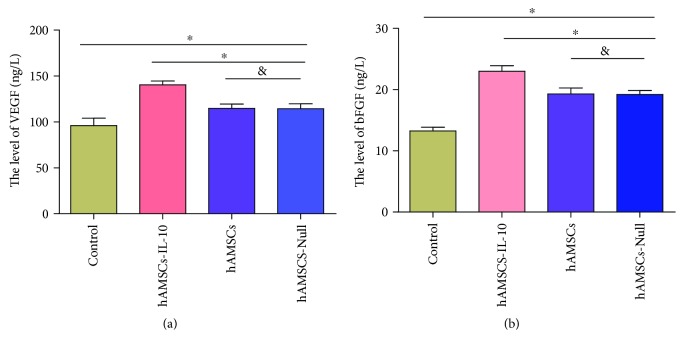
Expression of angiogenic factors on day 7 after cell transplantation by ELISA. ELISA showing comparison with controls: the hAMSCs-IL-10, hAMSCs, and hAMSCs-Null groups had higher expression, and hAMSCs-IL-10 had the highest expression of VEGF and bFGF. The hAMSCs-Null and hAMSC groups showed no significant differences (*n* = 3). ^∗^*P* < 0.05 and ^&^*P* > 0.05.

To determine the potential effect of hAMSCs-IL-10 on wound angiogenesis, the levels of angiogenic factors VEGF and bFGF in cutaneous wounds were examined by ELISA. The expression levels of VEGF and bFGF in hAMSCs-IL-10, hAMSCs, and hAMSCs-Null groups were significantly higher than that in the control group, and hAMSCs-IL-10 had the highest expression of VEGF and bFGF. The hAMSCs-Null and hAMSC groups showed no significant difference (Figure 7). These results indicated that hAMSCs-IL-10 increased wound angiogenesis.

### 3.6. IL-10-hAMSCs Enhanced Proper ECM Events during Healing

**Figure 8:**
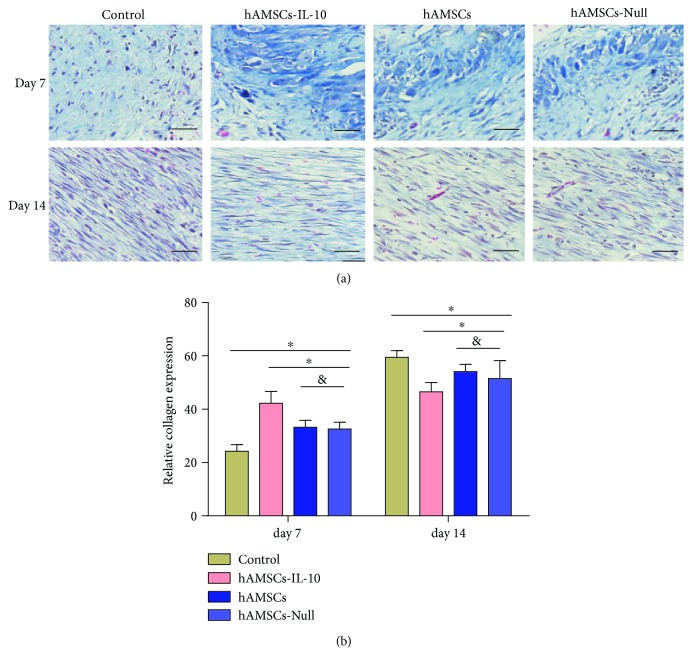
Collagen accumulation in wounds on days 7 and 14. (a) Masson staining of collagen deposition in the dermis showed increases in the hAMSCs-IL-10, hAMSCs, and hAMSCs-Null groups on day 7 compared with the control group; the hAMSCs-IL-10 group had the most collagen, and collagen was arranged regularly; the hAMSCs-Null and hAMSC groups were not significantly different. On day 14, the control group had the highest collagen deposition, and collagen was arranged irregularly. The hAMSCs-IL-10 group had the lowest collagen deposition. Scale bar = 50 *μ*m. (b) Quantification of collagen synthesis in wound skin by groups at indicated time points.^∗^*P* < 0.05 and ^&^*P* > 0.05.

ECM synthesis is an essential process of wound healing, and collagen is the main component of the ECM. To investigate the possible effect of hAMSCs-IL-10 on ECM production and remodeling during the healing process, accumulation of collagen was analyzed by Masson trichrome staining. All three hAMSC treatment groups had significant upregulation of collagen accumulation in skin compared with the control group on day 7 (Figures 8(a) and 8(b)). The IL-10-hAMSC group also showed higher collagen accumulation than the other groups, and collagen was arranged regularly. On day 14, collagen accumulation in the control group was significantly higher than in the other three groups, and collagen was arranged irregularly. The IL-10-hAMSC group showed a significantly lower collagen accumulation than the hAMSCs-Null and hAMSC groups.

**Figure 9:**
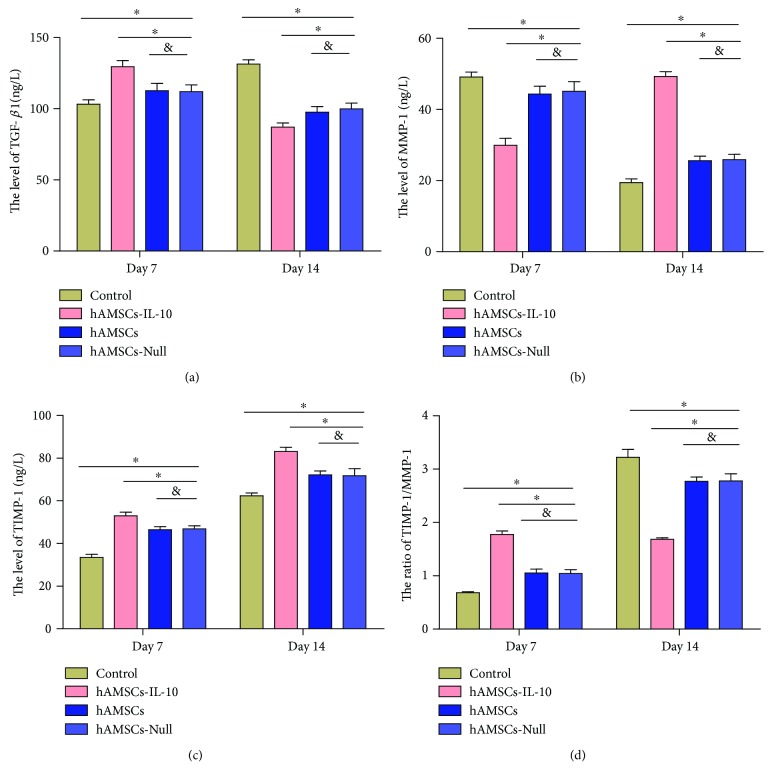
Effects of hAMSCs on wound ECM remodeling on days 7 and 14 after cell transplantation. (a) TGF-*β*1 expression by groups at indicated time points. The hAMSCs-IL-10 group showed the highest expression of TGF-*β*1 on day 7 compared with the hAMSCs, hAMSCs-Null, and control groups. On day 14, expression of TGF-*β*1 in the control group was significantly higher than that in the other groups with the hAMSCs-IL-10 group showing the lowest expression. (b) MMP-1 expression by groups at indicated time points. The hAMSCs-IL-10 group showed the lowest expression of MMP-1 on day 7 compared with the hAMSCs, hAMSCs-Null, and control groups. On day 14, expression of MMP-1 in the hAMSCs-IL-10 group was significantly higher than that in other groups. (c) TIMP-1 expression by groups at indicated time points. Expression of TIMP-1 was significantly increased at days 7 and 14 in wounds treated with hAMSCs-IL-10, hAMSCs, or hAMSCs-Null compared with the control group; the hAMSCs-IL-10 group showed the highest level. (d) TIMP-1/MMP-1 ratio was highest in the hAMSCs-IL-10 group on day 7 and lowest on day 14. ^∗^*P* < 0.05 and ^&^*P* > 0.05.

MMP and TIMPs are important in ECM remodeling. TGF-*β*1 regulates the expression of MMP-1 and TIMP-1. We used ELISA to analyze the expression of TGF-*β*1, MMP, and TIMPs at different time points after cell transplantation. Compared to the control group, the hAMSCs-IL-10, hAMSCs, and hAMSCs-Null groups showed significant upregulation of TGF-*β*1, and the hAMSCs-IL-10 group showed the highest expression on day 7 (Figure 9(a)). The results were reversed on day 14, and expression of TGF-*β*1 in the control group was significantly higher than that in the other three groups with the hAMSCs-IL-10 group showing the lowest expression (Figure 9(a)). On day 7, expression of MMP-1 in the hAMSCs-IL-10, hAMSCs, and hAMSCs-Null groups showed significant downregulation compared with that in the control group, and the hAMSCs-IL-10 group had the lowest level. This result was reversed on day 14, with the expression of MMP-1 in the hAMSCs-IL-10 group significantly higher that than in other groups (Figure 9(b)). The expression of TIMP-1 was significantly increased at days 7 and 14 in wounds treated with hAMSCs-IL-10, hAMSCs, or hAMSCs-Null compared with the control group, and the hAMSCs-IL-10 group showed the highest level (Figure 9(c)). The ratio of TIMP-1/MMP-1 was highest in the hAMSCs-IL-10 group on day 7 and lowest on day 14 (Figure 9(d)).

### 3.7. Colonization and Survival of hAMSCs *In Vivo*

**Figure 10:**
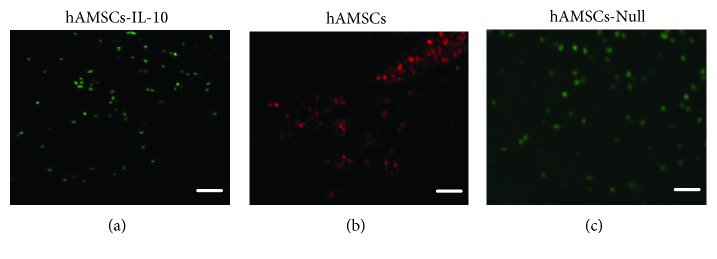
Fluorescent image of frozen sections of wound skin. When exposed to green light, transplanted hAMSCs showed red fluorescence. When exposed to blue light, hAMSCs-IL-10 and hAMSCs-Null expressed green fluorescence. Labeled cells concentrated in subcutaneous tissues on day 14 after cell transplantation, indicating that hAMSCs-IL-10, hAMSCs, and hAMSCs-Null survived after transplantation. Scale bar = 500 *μ*m.

To observe the colonization and survival of hAMSCs *in vivo*, hAMSCs were stained with CM-DiI, and hAMSCs-IL-10 and hAMSCs-Null expressed green fluorescence protein, 14 days after cell transplantation. Wound skin was fixed, and frozen sections were prepared and examined under fluorescence microscopy. Group fluorescence distributions indicated that hAMSCs, hAMSCs-IL-10, and hAMSCs-Null were colonized and survived in tissues (Figure 10).

## 4. Discussion

Although bone marrow stem cells (BMSCs) have been extensively studied for wound healing, their collection is associated with invasive procedures and amounts that are relatively low. The number of BMSCs falls as donor age increases [12]. The search for easily accessible and noninvasive procedures to obtain MSCs has focused on other human tissues, such as the placenta. hAMSCs are isolated from the amniotic membrane of human medical waste material with minimal ethical problems. Compared with MSCs from other sources, hAMSCs are isolated by simple enzymatic digestion procedures from a single amnion for more than 10^7^ cells with high proliferative capacity [13]. As hAMSCs express low levels of classical MHC-I and do not express MHC-II, they survive in immunocompatibility mismatched allogeneic transplant recipients. They are promising for applications in the field of regenerative medicine [14].

IL-10 has received attention because of its potent multiple biological effects. It is a pivotal factor in wound healing [8–10]. IL-10 delivery by scaffold materials such as collagen-silica [15], polycaprolactone (PCL) [16], transgene adjuvant D-mannose [17], and viruses [18] has been investigated. However, IL-10 has a short half-life *in vivo* [19]. Spatiotemporal control over bioactive molecule release can achieve optimal efficacy [19]. MSCs can regulate paracrine signals by sensing wound microenvironment, and IL-10 is a key factor in immunomodulatory properties of MSCs [20, 21]. In addition to having biological effects that promote wound regeneration healing, MSCs are an attractive vehicle for gene delivery for regeneration medicine [4, 6, 7]. Therefore, an excellent strategy would be MSCs as a vehicle for IL-10 delivery for wound regeneration healing. However, the biological effects of IL-10 delivery by MSCs in wound healing remain unclear.

In this study, we used the IL-10 gene to modify hAMSCs to exert synergistic effects such as modulating inflammation, enhancing angiogenesis, and regulating ECM remodeling, to promote regenerative wound healing. We found that hAMSCs-IL-10 secreted IL-10 at higher levels than unmodified hAMSCs. Wounds treated with hAMSCs-IL-10 showed more rapid wound closure and better wound-healing qualities.

Inflammation is an essential, nonspecific, innate immune response involving the breakdown of tissue and cleanup of cellular, extracellular, and pathogenic debris. However, in the presence of an external noxious stimulus that causes tissue damage, inflammation can become prolonged and heightened. IL-6 is a proinflammatory cytokine that exerts multiple effects in direct opposition to the anti-inflammatory cytokine IL-10, such as increasing inflammatory cell infiltration [22]. Evidence is provided by the formation of scars with addition of IL-6 in the fetus at a gestational age that should heal without scarring [23]. With IL-10 functions in fetal regenerative wound healing, these findings have led to the “cytokine hypothesis” that proposes that fetal tissue is permissive of regenerative healing due to relatively elevated levels of anti-inflammatory cytokine expression compared with proinflammatory cytokines, leading to an anti-inflammatory wound milieu [1, 2]. TNF-*α*, also an important proinflammatory cytokine, is one of the implicated molecules and has a key function in inflammation and subsequent wound healing [24]. Our results showed that hAMSCs-IL-10 attenuated the inflammatory response of local wounds by decreasing inflammatory cell infiltration as well as by production of proinflammatory cytokines IL-6 and TNF-*α*. hAMSCs-IL-10 administration clearly showed that anti-inflammatory effects were enhanced by synergistic effects of hAMSCs and IL-10.

VEGF is one of the most potent proangiogenic factors. It is secreted by keratinocytes and macrophages and acts to promote the proliferation of vascular endothelial cells [25]. bFGF is a strong angiogenic factor that stimulates the migration and proliferation of vascular endothelial cells and facilitates capillary formation. During wound healing, VEGF and bFGF synergistically stimulate endothelial cell proliferation, promote vascularization, and accelerate the process of wound healing [26]. Several studies demonstrated that MSCs in wound areas can secrete cytokines such as VEGF, bFGF, and PDGF, resulting in enhanced angiogenesis and wound healing. Some soluble factors secreted from MSCs induce endothelial cell survival, vascular branching, and pericyte recruitment [27]. Our study found that hAMSCs-IL-10 upregulated VEGF and bFGF and increased the density of microvessels in local wounds. We hypothesized that hAMSCs-IL-10 accelerated wound healing by paracrine VEGF and bFGF to increase wound angiogenesis.

TGF-*β*1 has multiple functions in the process of wound healing. First, TGF-*β*1 is involved in the regulation of the inflammation response. In the early stage of wound healing, the level of TGF-*β*1 is low, promoting the migration of neutrophils and macrophages. In the middle stage of wound healing, the level of TGF-*β*1 increases rapidly, which can suppress the migration and activation of lymphocytes and macrophages, and result in decreased inflammation [28]. Second, TGF-*β*1 is involved in the regulation of proliferation and differentiation of fibroblasts through autocrine and paracrine effects. Low concentrations of TGF-*β*1 promote proliferation and differentiation of fibroblasts and result in increased collagen deposition. High concentrations of TGF-*β*1 inhibit proliferation and differentiation of fibroblasts and decreased collagen deposition [28]. Third, TGF-*β*1 is involved in the regulation of MMP-1 and TIMP-1 in the ECM. MMP-1 is a major factor in the degradation of collagen in ECM. TIMP-1 is a special inhibitor of MMP-1. MMP-1 and TIMP-1 and constitutes a compact complex with proportion in dynamic equilibrium to regulate the degradation and deposition of ECM [29, 30]. High levels of TGF-*β*1 downregulate expression of MMP-1, resulting in an increase in TIMP-1 response. Eventually, the deposition of collagen increases and degradation of ECM reduces, resulting in scar hyperplasia [31]. Our results showed that hAMSCs upregulated the expression of TGF-*β*1 in the early and middle stages of wound healing and downregulated the expression of TGF-*β*1 in the middle and late stages of wound healing. In the middle and late stages of wound healing, hAMSCs increased the expression of MMP-1 and TIMP-1 and reduced the ratio of TIMP-1/MMP-1. By reducing inflammation and fibrosis, hAMSC administration accelerated wound healing and alleviated scar formation. IL-10 enhanced these effects of hAMSCs, and hAMSCs-IL-10 had stronger biological effects than hAMSCs.

hAMSCs-IL-10 transplantation promoted wound healing and improved healing qualities more effectively by upregulating expression of IL-10, modulating inflammation, enhancing angiogenesis, promoting granulation tissue formation, and regulating ECM remodeling. These data may thus provide a theoretical foundation for clinical administration of hAMSCs-IL-10 in cutaneous wound healing and skin regeneration. In this study, at 14 days after cell transplantation, we observed that cells were colonized and survived in tissues. However, the fate of hAMSCs after a long time is worth further investigation. Tracking and verification are complex and with remaining questions to be answered, such as cell fusion before the final destination becomes clear.

## Data Availability

The data used to support the findings of this study are included within the article.
